# Semi-Interpenetrated Polymeric Networks of Chitosan and Poly(γ-Glutamic Acid) with Potential Biomedical Applications

**DOI:** 10.1021/acsomega.5c13458

**Published:** 2026-03-03

**Authors:** Yaniris Garmendía-Diago, Dora Evelia Rodríguez-Félix, María Mónica Castillo-Ortega, Teresa Del Castillo-Castro, Francisco Rodríguez-Félix, Juana Alvarado-Ibarra, Lerma Hanay Chan-Chan, Irela Santos-Sauceda, José Ramón Flores-León, Antonio Serguei Ledezma Pérez

**Affiliations:** † Departamento de Investigación en Polímeros y Materiales, 27813 Universidad de Sonora, Hermosillo 83000, Sonora, Mexico; ‡ Departamento de Investigación y Posgrado en Alimentos, Universidad de Sonora, Hermosillo 83000, Sonora, Mexico; § Departamento de Física, Universidad de Sonora, Hermosillo 83000, Sonora, Mexico; ∥ Centro de Investigación en Química Aplicada, Saltillo 25294, Coahuila, Mexico

## Abstract

Hydrogels are polymeric matrices very similar to living tissue due to their elasticity, porosity, and ability to absorb high water content. They are highly attractive materials for a wide range of biomedical applications, such as tissue engineering, wound healing, and drug delivery. In this regard, hydrogels of semi-interpenetrating polymer networks (semi-IPNs) based on the biopolymers chitosan and poly­(γ-glutamic acid) (γ-PGA) were prepared as systems with improved properties compared to hydrogels of individual polymers. The resulting hydrogels were characterized by Fourier transform infrared spectroscopy (FTIR), scanning electron microscopy (SEM), thermogravimetric analysis (TGA), porosity testing, compressive strength, cell viability, and swelling capacity. FTIR spectra of the semi-IPNs confirmed the presence of the functional groups of each polymer. SEM images revealed a porous structure of the hydrogels, which became denser and more compact with increasing γ-PGA content. This behavior was corroborated by the porosity test, which decreased with the formation of the γ-PGA-reinforced network. The swelling capacity study of the hydrogels demonstrated their sensitivity to pH and temperature. For the semi-IPN hydrogels, SF1 had the highest swelling ratio (20.55) at pH 3.6 and *T* = 37 °C. The formation of the semi-IPNs brought about improvements in mechanical properties compared to the chitosan hydrogel. The presence of γ-PGA contributed to improved biocompatibility of the materials, especially in formulations with 0.025 and 0.05 g of this biopolymer. These results suggest that the obtained chitosan/γ-PGA semi-interpenetrating networks may be promising materials with great potential for use in biomedical applications.

## Graphical abstract

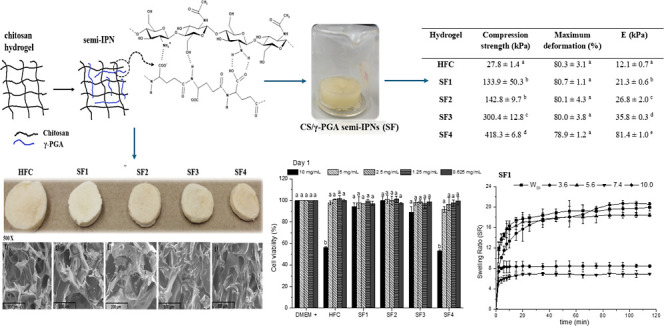

## 1 Introduction

Hydrogels are a category of soft and moist polymeric materials characterized by a three-dimensional interconnected polymeric network that incorporates significant quantities of water as the filled solvent.^1^ The hydrophilic functionalities, including: –NH_2_, –CONH, –SO_3_H, –CONH_2_, –COOH, and –OH, are responsible for holding a large amount of water. The cross-linking of network chains facilitates this water retention, enabling the structures to maintain their integrity without dissolution.^2–4^ Most hydrogels exhibit biocompatibility and a high water content, making their properties comparable to those of soft tissues such as skin, tendons, and cartilage.^1,5^ The prevailing trend in the development of this type of material involves the formulation of hydrogels that demonstrate the ability to respond to a range of stimulus, including pH, temperature, biological molecules, electric fields, magnetic fields, and mechanical forces. This responsiveness characterizes them as “smart” biomedical materials, suitable for use in advanced medical devices.^6,7^ However, conventional hydrogels often exhibit weak mechanical properties due to their low polymer density, heterogeneous network structures, and limited friction between polymer chains, significantly restricting their applicability in load-bearing conditions.^1^ Thus, there has been a significant increase in research focus on interpenetrating polymer networks (IPNs).^8^ Interpenetrating polymer networks constitute a class of hydrogels formed by two or more interwoven polymer networks that do not involve the establishment of covalent bonds.^9^ IPNs can be classified into sequential, simultaneous, and semi-interpenetrating types. Semi-interpenetrating gels are created by enclosing a linear polymer within another polymer network without the formation of chemical bonds. This combination of polymers results in an advanced multicomponent polymeric system that leverages the distinct characteristics and properties of each network or polymer. Therefore, these properties are enhanced, leading to the formation of a more effective reinforced system.^10–14^ Consequently, there is an increasing interest in utilizing biopolymer-based interpenetrating polymer networks (IPNs) for different biomedical applications.^15–20^ Chitosan and poly­(γ-glutamic acid) are two biopolymers that, due to their excellent and unique characteristics and properties, represent a promising alternative for the design and preparation of novel semi-interpenetrating polymer networks (semi-IPNs) for biomedical applications.^11^

Chitosan (CS) is a polysaccharide widely used for biomedical applications including tissue engineering and drug delivery, approved by the Food and Drug Administration (FDA). It is obtained through partial alkaline deacetylation of chitin, found in the exoskeletons of crustaceans and insects, and the cell walls of fungi.^21–24^ Structurally, it is a polysaccharide constituted by *N*-glucosamine and *N*-acetylglucosamine units, in which the number of *N*-glucosamine units exceeds 50%.^25^ Chitosan has several advantageous properties such as biocompatibility, biodegradability, antimicrobial activity, cell adhesion behaviors, and nontoxicity.^21,24–27^

Poly­(γ-glutamic acid) (γ-PGA) is an anionic homopolyamide composed of D- and l- glutamic acid units connected by γ-amide linkages between α-amino and γ-carboxyl groups. It is synthesized via secretion into the extracellular matrix by various microbial strains, especially *Bacillus* species.^28–30^ γ-PGA is at present receiving great attention due to its enormous possibilities as a biomaterial because it is biodegradable, nontoxic, biocompatible, and even edible.^31–35^ In addition, in its free-acid form, γ-PGA can be chemically cross-linked, producing biohydrogels.

Than-ardna et al. synthesized semi-IPNs based on chitosan and poly­(2-hydroxyethyl methacrylate) (CS/PHEMA) through free radical polymerization of HEMA in the presence of CS solution with potassium persulfate (KPS) as an initiator and triethylene glycol dimethacrylate (TEGDMA) as a cross-linking agent.^36^ Wahid et al. prepared semi-IPNs hydrogels based on bacterial cellulose (BC) and chitosan (CS), by blending BC’s slurry with CS solution and cross-linking with glutaraldehyde. The polymers were associated with better mechanical properties and possibly synergistic combinations of the properties of their components.^37^ Zhu et al. synthesized pH-sensitive semi-IPN hydrogels by using konjac glucomannan (KGM) with sodium trimetaphosphate (STMP) as the cross-linking agent, and γ-PGA, as a potential biomaterial for drug delivery in the intestine.^38^ Dou et al. synthesized a double-network (DN) full biological hydrogel with excellent mechanical properties and biocompatibility for wound healing, by introducing a physically cross-linked gelatin (GEL) network in a covalently cross-linked γ-PGA network with ethylene glycol diglycidyl ether (EGDE).^39^

In general, these studies demonstrate promising properties; however, our objective is to develop novel semi-IPN hydrogel systems based on two biopolymers with highly desirable characteristics, such as chitosan and γ-PGA, using a simple fabrication process involving autoclaving and freezing, thereby avoiding the use of toxic cross-linking agents.

In the present work, we report the synthesis of semi-IPNs, based on chitosan and γ-PGA, as well as the physicochemical characterizations performed, including assessments of mechanical properties, swelling capacity, and biocompatibility. The purpose of our research is to develop a biomaterial with promising properties and attributes for biomedical applications, such as controlled drug delivery systems and tissue engineering.

## 2 Materials and Methods

### 2.1 Materials

Glacial acetic acid 99.5% was purchased from FAGA LAB. Chitosan- medium molecular weight (*M*_w_ = 190–310 kDa; deacetylation ≥75%) was purchased from Sigma-Aldrich. Pure water was obtained from a Milli-Q Plus water purification system (Millipore, Advantage A10). All reagents were used as received. Poly­(γ-glutamic acid) (*M*_w_ = 260,000 g/mol; PDI = 1.77) from *Bacillus licheniformis* (ATCC 9945a) was obtained in the laboratory according to a procedure described in a previous study.^40^

### 2.2 Preparation of Chitosan Physical Hydrogel

The hydrogel was prepared by dissolving 0.1 g of chitosan in 4 mL of acetic acid 1% (v/v). It was stirred at 300 rpm at 50 °C for 10 min until the chitosan was completely dissolved. The polymer solution was placed in an autoclave at 121 °C for 30 min. It was then allowed to cool to room temperature and frozen for 24 h. After this time, the hydrogel was thawed at room temperature. The physical hydrogel formed was washed three consecutive times every 30 min with deionized water. Finally, the hydrogel was frozen and dried using a Labconco Freezone 4.5 freeze-dryer.

### 2.3 Preparation of Semi-IPN Networks Hydrogels of Chitosan and γ-PGA

Four different semi-IPNs were prepared by varying the mass of γ-PGA, as shown in Table 1. γ-PGA solutions with different biopolymer contents (as specified in Table 1) were prepared in 2 mL of deionized water, maintaining constant magnetic stirring at 300 rpm and room temperature for 10 min. Subsequently, the chitosan physical hydrogel was immersed in the γ-PGA solution, absorbing it completely. The hydrogel was allowed to rest for 24 h for complete absorption of the solution and was subsequently frozen and freeze-dried.

**Table 1:** Composition of the Hydrogels According to the Proportions by Weight of the Polymers

hydrogel	chitosan (g)	γ-PGA (g)	*w* _chitosan_: *w* _γ‑PGA_
HFC	0.1	0	1:0
SF1	0.1	0.025	1:0.25
SF2	0.1	0.05	1:0.5
SF3	0.1	0.075	1:0.75
SF4	0.1	0.1	1:1

### 2.4 Characterization by Infrared Spectroscopy (FT-IR)

The structural analysis of the simple chitosan hydrogel and the semi-IPNs networks was carried out using a PerkinElmer spectrophotometer, Frontier model, by the attenuated total reflectance (ATR) technique. The analysis of the samples was carried out in a scan of the infrared spectrum from 4000 to 400 cm^–1^. This study was realized to identify the functional groups that must be present in each material.

### 2.5 Characterization by Scanning Electron Microscopy

The morphological analysis of the chitosan hydrogel and the semi-IPNs networks was carried out using a JEOL 5410LV scanning electron microscope. The samples were coated with gold to give them conductive properties and a 15 kV intensity electron beam was used. The pore size of the hydrogels was determined using the ImageJ program. The size distribution was determined using the OriginLab 2022b program.

### 2.6 Determination of the Percentage of Porosity

The percentage porosity of the hydrogels was determined by weighing the samples in the dry state. Then, the hydrogels were placed in 20 mL of anhydrous ethanol for 30 min. Subsequently, the samples were extracted, and excess alcohol was removed using filter paper. Finally, the treated hydrogel samples were weighed. The percentage of porosity was calculated using the following equation (eq 1)­

1 $$Porosity\left(\%\right)=\frac{\left(M_{2}-M_{1}\right)}{\rho \times V}\times 100$$

where *M*_1_ is the initial weight of the hydrogel in the dry state, *M*_2_ is the weight of the hydrogel after saturation with anhydrous alcohol, ρ is the density of absolute ethanol (0.789 g/mL), and *V* is the volume of the samples of hydrogel in the dry state. All samples were analyzed in triplicate.^41–45^

### 2.7 Compression Test

The mechanical compression resistance tests of the hydrogels were performed using micromechanical equipment (ElectroForce 5110, USA), at room temperature. For the compression test, a maximum load cell of 200 N was used at a constant speed of 1 mm/s. The hydrogel samples analyzed had a cylindrical shape (10 ± 2 mm diameter, 10 ± 1 mm height) and were swollen to pH = 5.6 (simulating the skin’s pH). The elastic modulus of the samples was calculated from the stress–strain curves. All tests were performed in quintuplicate.

### 2.8 Thermogravimetric Analysis

Thermogravimetric analysis of γ-PGA, chitosan hydrogel, and semi-IPNs hydrogels was performed. A Thermogravimetric Analyzer, TermoFisher Scientific TGA 600, was used. Samples of approximately 5 mg were weighed and subjected to heating from 25 to 700 °C, with a heating rate of 10 °C/min in a nitrogen atmosphere.

### 2.9 Swelling Study of Hydrogels

The swelling ratio of the chitosan hydrogel and the semi-IPN hydrogels was determined by examining the effects of pH and temperature. Dried hydrogel samples measuring 5 mm in diameter and 5 mm in thickness were employed in this study. Deionized water and buffer solutions with pH values of 3.6, 5.6, 7.4, and 10.0 were used as swelling mediums. The dried hydrogel samples were immersed in containers containing 20 mL of the respective swelling medium at 25 and 37 °C. The weight of the hydrogels was monitored at 10 min time intervals. The hydrogels were weighed on a Sartorius analytical balance (sensitivity 0.0001 g) and previously dried with filter paper to eliminate water on the surface of the sample. This procedure was carried out until equilibrium swelling was reached. The study was carried out in triplicate, and the values reported are the average values of the three measurements in each condition. The swelling ratio was calculated using eq 2

2 $$Swelling\;Ratio\left(SR\right)=\frac{M_{s}-M_{d}}{M_{d}}$$

where *M*s is the weight of the swollen gel at different times, and *M*d is the weight of the dry gel.^46–48^

### 2.10 In Vitro Cytotoxicity Assay

The cytocompatibility of the hydrogels was estimated by the Resazurin reduction assay. The viability of the human embryonic fibroblast cell line Detroit 548 CCL 116 was evaluated. The assay was carried out by the indirect noncontact method (extracts or dilutions), according to the procedure described in ISO 10993-5: 2009. The hydrogels were sterilized for 30 min on both sides under ultraviolet light and then the leaching solutions (10 mg/mL) were obtained by immersing the sterile hydrogels in the Dulbecco’s Modified Eagle’s Medium (DMEM) supplemented with fetal bovine serum (Gibco) at 5%, antibiotics 1% (10 mg/mL streptomycin, 10^4^ U penicillin, Sigma-Aldrich). Four dilutions were made to the extract solution, at the concentrations of 5, 2.5, 1.25, and 0.625 mg/mL. Cells were seeded in a 96-well plate at an initial density of 10^4^ cells per well in the supplemented DMEM medium and incubated for 1 h, at 37 °C, 5% CO_2,_ and 80% humidity. After the incubation time had elapsed, the culture medium was removed, leaving the cells attached to the bottom of the plate. Then, 100 μL of each of the extracts and their respective dilutions were added to the plate. The wells containing only the supplemented DMEM medium were set as control groups. Subsequently, the cells were incubated under the same conditions mentioned above for 3 consecutive days. On each of the incubation days, the culture medium was removed from each well and 100 μL of a resazurin solution with a concentration of 0.02 mg/mL was added and incubated for 8 h. Absorbance at 570 and 600 nm was measured on each plate using a Synergy HTX Multi-Modal Microplate Reader. Finally, the percentage of cell viability was determined by eq 3

3 $$Cell\;viability\left(\%\right)=\frac{\left(O_{2}\times {\;A}_{1}\right)-\left(O_{1}\times {\;A}_{2}\right)}{\left(O_{2\;}\times {\;P}_{1}\right)-\left(O_{1}\times P_{2}\right)}\times 100$$

where *O*_1_ is the molar extinction coefficient (*E*) of oxidized Alamar Blue at 570 nm, *O*_2_ is the *E* of oxidized Alamar Blue at 600 nm, *A*_1_ is the absorbance of test wells at 570 nm, *A*_2_ is the absorbance of test wells at 600 nm, *P*_1_ is the absorbance of positive growth control well at 570 nm and *P*_2_ is the absorbance of positive growth control well at 600 nm.^49^

### 2.11 Statistical Analysis

Values reported in each experiment were presented as mean ± SD. Statistical difference was determined by two-way analysis of variance (ANOVA) followed by multiple comparison procedures using Tukey’s method. The minimal level of significance was taken as *p* < 0.05. The letters above the error bars indicate significance: different letters indicate the existence of significant differences between the means of the groups compared; identical letters suggest that there are no significant differences.

## 3 Results and Discussion

### 3.1 Chitosan Physical Hydrogel and Chitosan/γ-PGA Semi-IPNs

Figure 1 illustrates the chitosan physical hydrogel, which is macroscopically observed as a light-yellow hydrogel exhibiting good structural stability, along with flexibility and ease of manipulation (Figure 1c,d). The formation of this network was achieved by the autoclave heating method, through which a complete dissolution of the polymer and a condensed state of the polysaccharide solution is achieved (Figure 1a). Subsequently, the second stage consists of a freezing-thawing process (Figure 1b). This technique is used for physical hydrogels based on polysaccharides in the absence of organic solvents and toxic cross-linkers. In the freezing process, cooling to subzero temperature induces liquid–liquid phase separations and promotes the transformation of water into ice in a polymer-poor phase. These ice crystals eject segments of amorphous polysaccharides, and bulk water crystallization reduces the available space occupied by the polysaccharide chains, increasing polymer concentrations, and thus the chains of polysaccharide molecules are forced to associate linearly and laterally in liquid microphase through noncovalent interactions between functional groups (carboxyl, hydroxyl, and amino). Upon thawing, cross-linked polysaccharide chains constitute the polymeric structure of the hydrogel. The entire structure of the hydrogel is stabilized mainly by the multiple and coexisting inter- and intramolecular hydrogen bonds in the junction zones of the polymer network. Under freeze-thaw treatment, the molecular chains of these polysaccharides can form ordered structures known as microcrystalline zones, which function as physical binding nodes in the network. The intermolecular bonds in these junction nodes are mainly hydrogen bonds that were formed between the hydroxyl, carboxylic, and amino groups in the polysaccharide backbones.^50,51^

**Figure 1:**
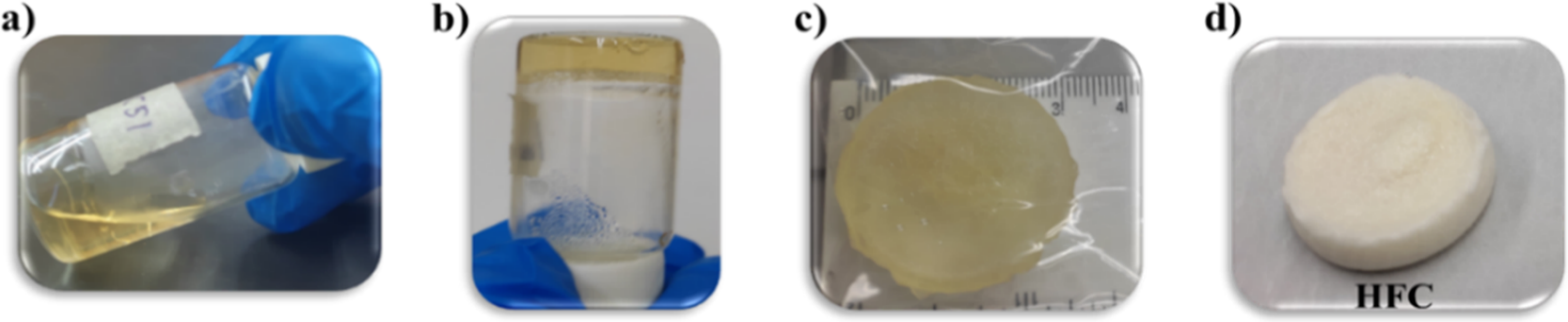
(a,b) Images of the chitosan physical hydrogel (HFC), before and after the gelation process, respectively; (c) HFC in its hydrated state; (d) HFC xerogel.

Macroscopically, the physical characteristics of the chitosan hydrogel show a light yellow color and easy manipulation (Figure 2c). The starting point was the chitosan hydrogel obtained previously. Subsequently, this hydrogel absorbs a solution of γ-PGA (Figure 2b), and the linear chains of this polymer intertwine within the physical network of chitosan, establishing physical interactions (Figure 2a) such as electrostatics and hydrogen bonds between both biopolymers.

**Figure 2:**
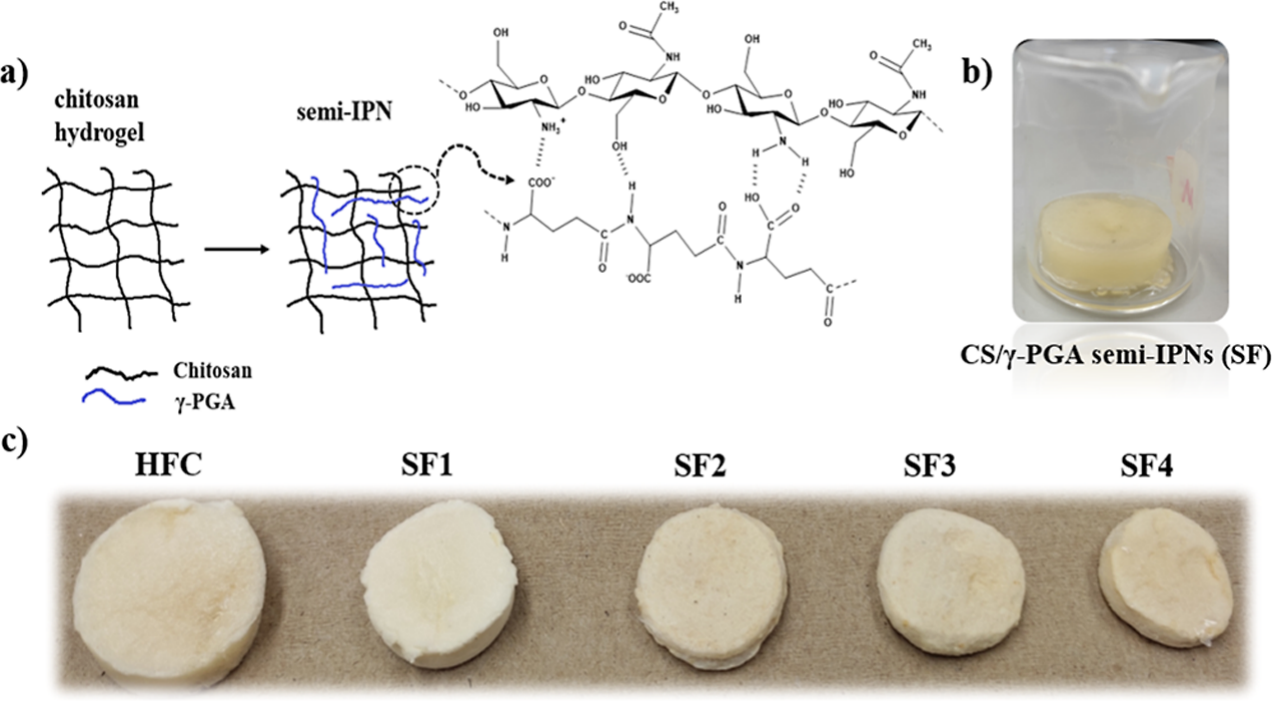
(a) Formation of semi-IPNs by physical interactions between chitosan and γ-PGA; (b) HFC hydrogel swollen in a γ-PGA solution; (c) images of the xerogels at the different proportions of γ-PGA.

### 3.2 Characterization by Infrared Spectroscopy (FT-IR)

FT-IR structural analysis of γ-PGA, chitosan hydrogel, and chitosan/γ-PGA semi-IPNs was performed. Comparisons were made between the spectra (Figure 3) of the different samples to assess whether the semi-IPNs were formed. A broadband between 2500 and 3500 cm^–1^ was observed in all spectra, attributed to the overlap of the stretching vibration signals of the –OH and –NH– groups present in the polymers. The spectrum of the chitosan hydrogel shows the characteristic signals of Amide I and II corresponding to the acetylated units at 1653 and 1597 cm^–1^, and a broadband around 1088 cm^–1^ associated with the stretching vibration ν (C–O–C), which is characteristic of the pyranosic ring, present in chitosan. In the γ-PGA spectrum, the signals corresponding to the carbonyl bond (C=O) of the lateral carboxyl group (–COOH), Amide I (ν_C=O_ of the –NH–CO- group), and Amide II (δ _N_–_H_) were observed in 1716, 1618, and 1530 cm^–1^, respectively. The formation of the semi-IPNs can be inferred by the presence in their spectra of the characteristic signals of both polymers. Furthermore, it was observed that by increasing the γ-PGA content, from SF1 to SF4, the bands corresponding to Amides I and II widen. In addition, displacements in the signals of the semi-IPNs were observed regarding the spectrum of chitosan hydrogel and the spectrum of γ-PGA. This behavior can be associated with the presence of γ-PGA, which indicates the existence of interactions between both polymers.^32,40,52^

**Figure 3:**
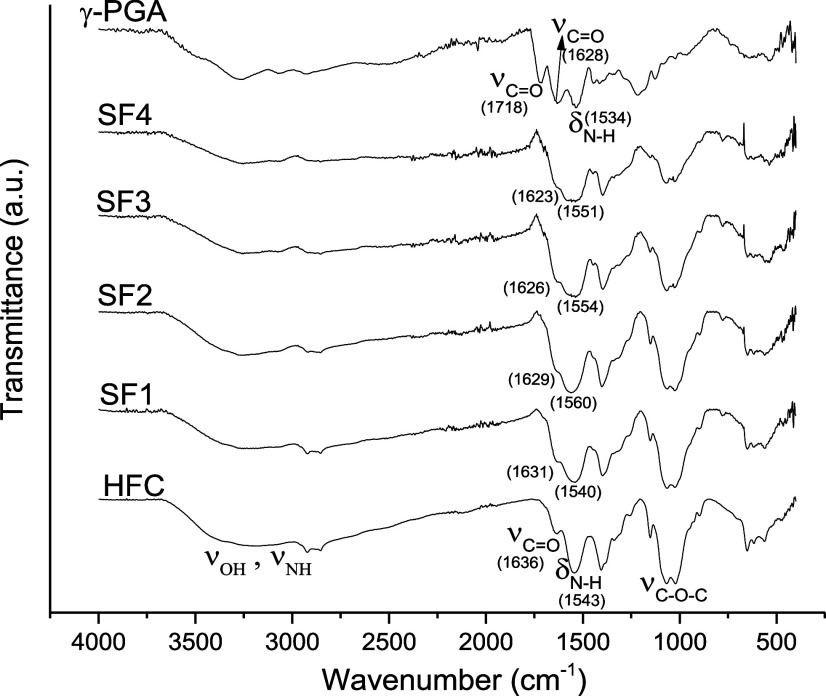
IR spectra of chitosan hydrogel, semi-IPNs (chitosan/ γ-PGA) and γ-PGA.

### 3.3 Scanning Electron Microscopy

Figure 4 shows the micrographs obtained from the cross-section of the hydrogels and the distribution of pore sizes. SEM images reveal a porous morphology in all hydrogel samples. The hydrogels presented an average pore size of 47.2 ± 6.2 μm, 57.0 ± 8.9 μm, 59.1 ± 5.9 μm, 49.6 ± 4.2 μm, and 46.8 ± 6.2 μm for HFC, SF1, SF2, SF3, and SF4, respectively. The porous morphology presented is a structural characteristic of hydrogels that gives them a potential capacity to store solvents inside, which is very useful for biomedical applications.^8,27^ In the semi-IPNs (SF1–SF4), increasing γ-PGA content reveals a more heterogeneous morphology with more irregular pores. An increase in the diameter of some pores and a lower number of pores per unit area are observed. The semi-IPNs become denser and more compact as the γ-PGA content increases. These behaviors may be related to the presence and increase in γ-PGA content, leading to increased interactions between both polymers. In this way, the physical cross-linking points increase, causing the polymer chains to become closer and more ordered, generating a matrix with thicker walls. This behavior has also been reported by Bhumin Than-ardna et al., who prepared semi-IPNs based on chitosan and poly­(2-hydroxyethyl methacrylate) (CS/PHEMA).^36^ On the other hand, Özbaş and Gürdaǧ, mentioned that in the formation of semi-IPN networks based on chitosan (CS) with acrylamide (AAm) and/or *N*-hydroxymethylacrylamide (HMA), there was a considerable decrease in the porosity of the simple hydrogel of chitosan when HMA was incorporated into the network, which was consistent with the swelling values they obtained.^53^ Suo et al., formed interpenetrated networks (IPNs) of gelatin methacryloyl (GelMA)/CS and proposed that the semi-IPN structure shows a much denser structure with smaller pores than those of the pure CS and GelMA hydrogels. Furthermore, as the CS concentration increases, larger pores with thicker pore walls are formed in IPNs, indicating that more macromolecular chains are intertwined and linked together.^54^

**Figure 4:**
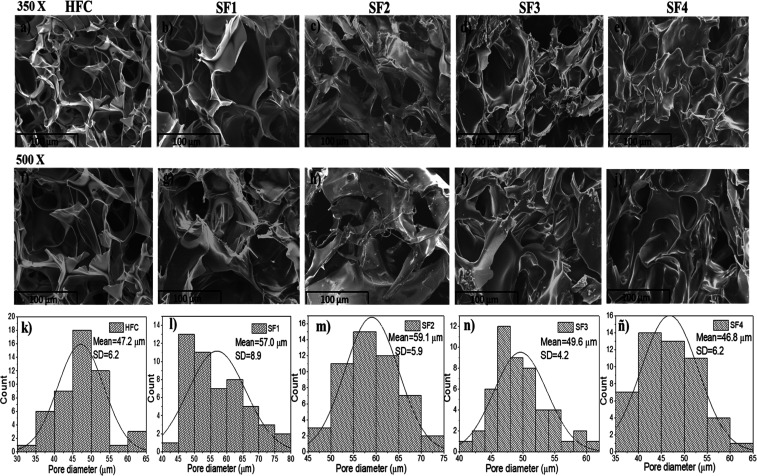
SEM images at 350X, 500X of the hydrogels: (a,f) HFC; (b,g) SF1; (c,h) SF2; (d,i) SF3, and (e,j) SF4; respectively. Pore diameter distribution: (k–n), and (ñ) of HFC, SF1, SF2, SF3, and SF4, respectively.

### 3.4 Porosity Percentage of Hydrogels

Figure 5 shows the graph of the percentage of porosity of hydrogels. The percentage porosity values were 29.2 ± 0.1%, 27.5 ± 1.4%, 19.0 ± 1.7%, 16.2 ± 0.9% and 15.1 ± 0.9% for HFC, SF1, SF2, SF3 and SF4, respectively. The porosity percentage decreases significantly for hydrogels SF2, SF3, and SF4 concerning HFC and SF1. The porosity decreased with increasing interconnected γ-PGA content. This trend can be attributed to the increased molecular entanglements between the CS network and γ-PGA chains due to higher physical cross-linking density, resulting in less pore formation between the polymers, leading to a decrease in the porosity of the hydrogel matrix, the formation of thick walls, and the formation of more compact networks. The results obtained by SEM corroborate this effect. This behavior was also presented by the semi-IPNs (CS/PHEMA) reported by Bhumin Than-ardna et al.^36^ Ziwei Hu et al. prepared CS/β-Ala/γ-PGA hydrogels and reported that the porosity of the hydrogels was negatively correlated with the degree of cross-linking and mechanical properties.^41^

**Figure 5:**
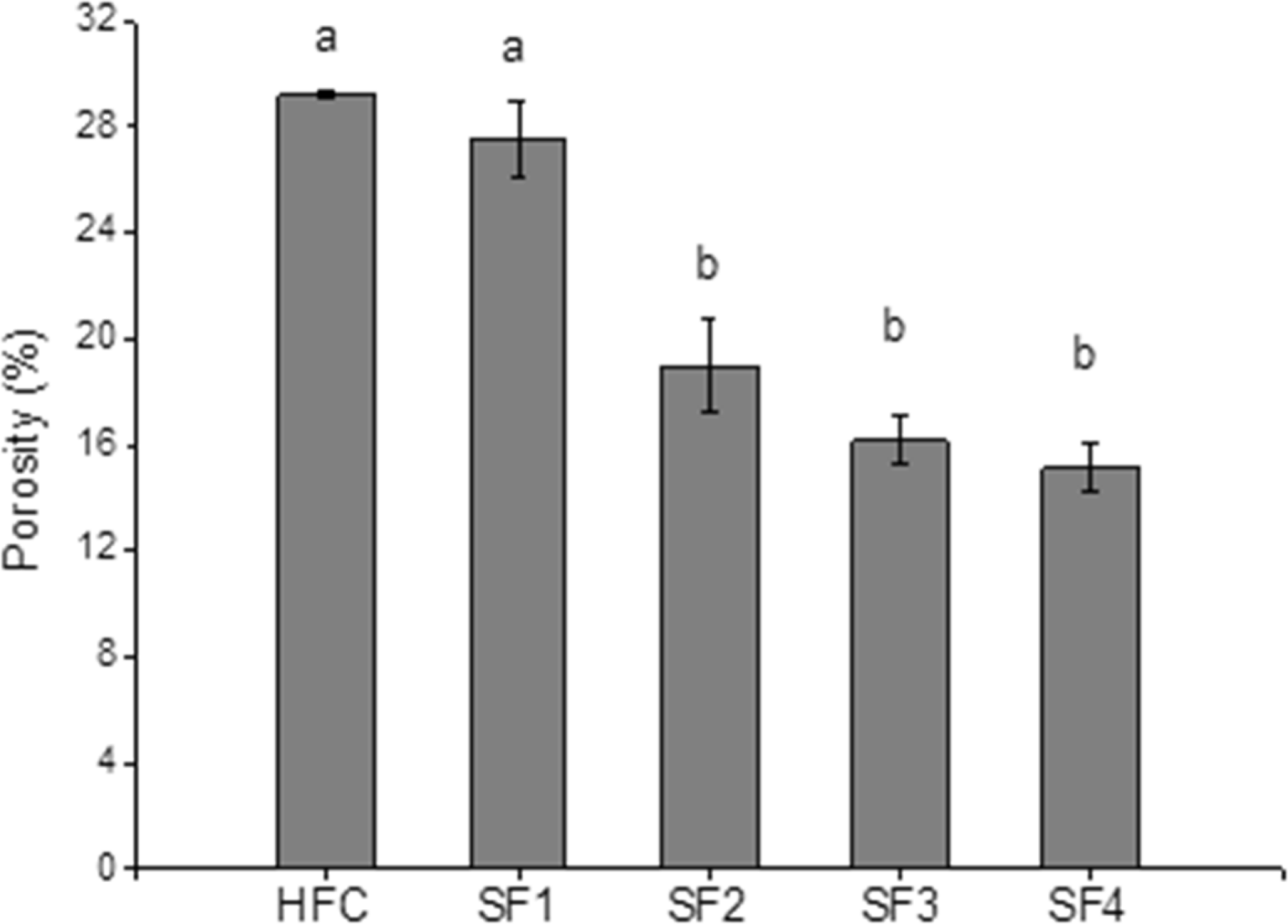
Porosity graph (%) of chitosan hydrogel and semi-IPNs (chitosan/ γ-PGA).

### 3.5 Compression Resistance Tests

The mechanical properties of the hydrogels were evaluated at compressive stress. The values obtained for maximum strength, maximum deformation, and the Modulus of elasticity (*E*) were reported in Table 2. For all samples, the experiment was stopped when the separation between the plates reached the limit. The semi-IPNs were not fractured. All hydrogels could recover to the applied stress, with the recovery speed being greater as the γ-PGA content increased. The maximum compressive strength and elastic modulus increase with increasing γ-PGA content, increasing its rigidity. The hydrogels had a high percentage of deformation without fracturing, showing good elasticity properties. The results obtained indicate that the presence of γ-PGA improves the mechanical properties of hydrogel. The compressive stress (CSS) in IPNs usually exhibit values ranging between 0.04 and 4.01 MPa, which are generally higher than those of the corresponding simple networks (SN) (0.02 MPa–2.5 MPa).^55^ Therefore, the hydrogels obtained present adequate mechanical properties consistent with those reported in the literature for hydrogel systems based on the biopolymers used in our study. Suo et al., reported physical chitosan hydrogels with a compression modulus of 3.43 ± 0.87 kPa.^54^ Bajestani et al., synthesized γ-PGA-Keratin chemical hydrogels with a compression modulus of the wet samples of 2900 Pa;^56^ Chen et al. prepared an alginate-chitosan chemical hydrogel with a maximum compression modulus of 9.21 kPa.^57^ Than-ardna et al., prepared CS/PHEMA semi-IPNs in the wet state and presented maximum compressive strength of 22.23 ± 2.19 to 25.86 ± 2.35 kPa.^36^ Based on the above, we can deduce that the prepared chitosan and γ-PGA hydrogels present good mechanical properties.

**Table 2:** Mechanical Properties of Hydrogels to Compressive Stress

hydrogel	compression strength (kPa)	maximum deformation (%)	*E* (kPa)
**HFC**	27.8 ± 1.4^a^	80.3 ± 3.1^a^	12.1 ± 0.7^a^
**SF1**	133.9 ± 50.3^b^	80.7 ± 1.1^a^	21.3 ± 0.6^b^
**SF2**	142.8 ± 9.7^b^	80.1 ± 4.3^a^	26.8 ± 2.0^c^
**SF3**	300.4 ± 12.8^c^	80.0 ± 3.8^a^	35.8 ± 0.3^d^
**SF4**	418.3 ± 6.8^d^	78.9 ± 1.2^a^	81.4 ± 1.0^e^

### 3.6 Thermogravimetric Analysis

The thermal stability of the hydrogels was evaluated by thermogravimetric analysis. The thermograms (Figure 6) revealed the first weight losses in the temperature range: *T* = 39.65–146.07 °C, corresponding to the water loss of the samples. The derivatives of the thermogravimetric curves show that the thermal degradation of γ-PGA and HFC, SF1, SF2, SF3, SF4 hydrogels initiates at 250.06, 274.16, 272.68, 264.56, 237.76, and 235.06 °C, respectively. For γ-PGA, the first step of thermal degradation starts above 200 °C according to an end-chain decompression mechanism, generating pyroglutamic acid and methyl pyroglutamate, followed by a cyclodepolymerization of the polymer chain.^58^ For the chitosan hydrogel (HFC), the first weight losses (15.21%) correspond to the evaporation of the water physically absorbed and bound to the chitosan by hydrogen bonds. Its thermal decomposition started by the depolymerization of the chains through the cleavage of glycosidic bonds and deacetylation. In the last stages, for temperatures above 400 °C (weight loss of 14.72%), it corresponds to the thermal degradation of the pyranotic ring and the decomposition of the residual carbon.^59^ Semi-IPNs hydrogels showed an intermediate thermal stability between chitosan hydrogel (HFC) and (γ-PGA). The semi-IPNs with higher chitosan content degraded at a higher temperature compared to the semi-IPNs with a higher proportion of γ-PGA. Therefore, we can say that chitosan contributes to improving the thermal stability of hydrogels.

**Figure 6:**
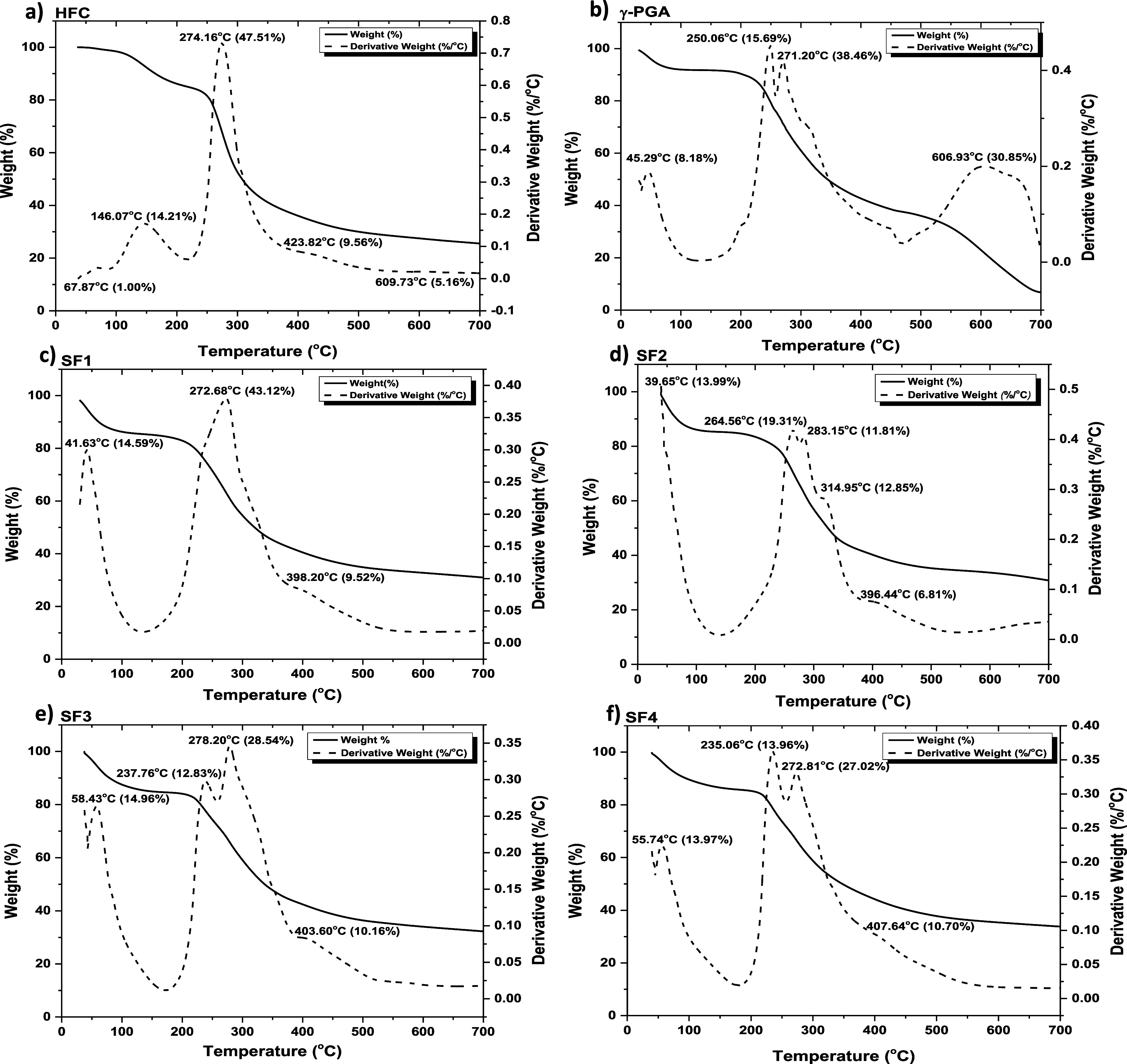
TGA and DTG thermograms: (a) HFC, (b) γ-PGA, (c) SF1, (d) SF2, (e) SF3, and (f) SF4, respectively.

### 3.7 Swelling Capacity of Hydrogels

The results obtained from the study of the swelling kinetics of the hydrogels at 37 and 25 °C are shown in Figures 7 and 8, respectively. The swelling ratio values are reported in Table 3. Relatively higher values can be observed at 37 °C compared to those obtained at 25 °C in most cases. The effect of temperature on the swelling of hydrogels can be explained by the formation and dissociation of hydrogen bonds between the polymer chains. As the temperature of the swelling medium increases, the hydrogen bonds between the hydrophilic groups in the polymer chains break down, which causes separation between them, facilitating the diffusion of water into the matrix. On the other hand, by dissociating the hydrogen bonds, a greater number of free sites that interact with water are produced, enabling the hydrogel to accept a greater amount of water within the polymeric network, which increases the swelling of the hydrogel.^46–48,54^

**Figure 7:**
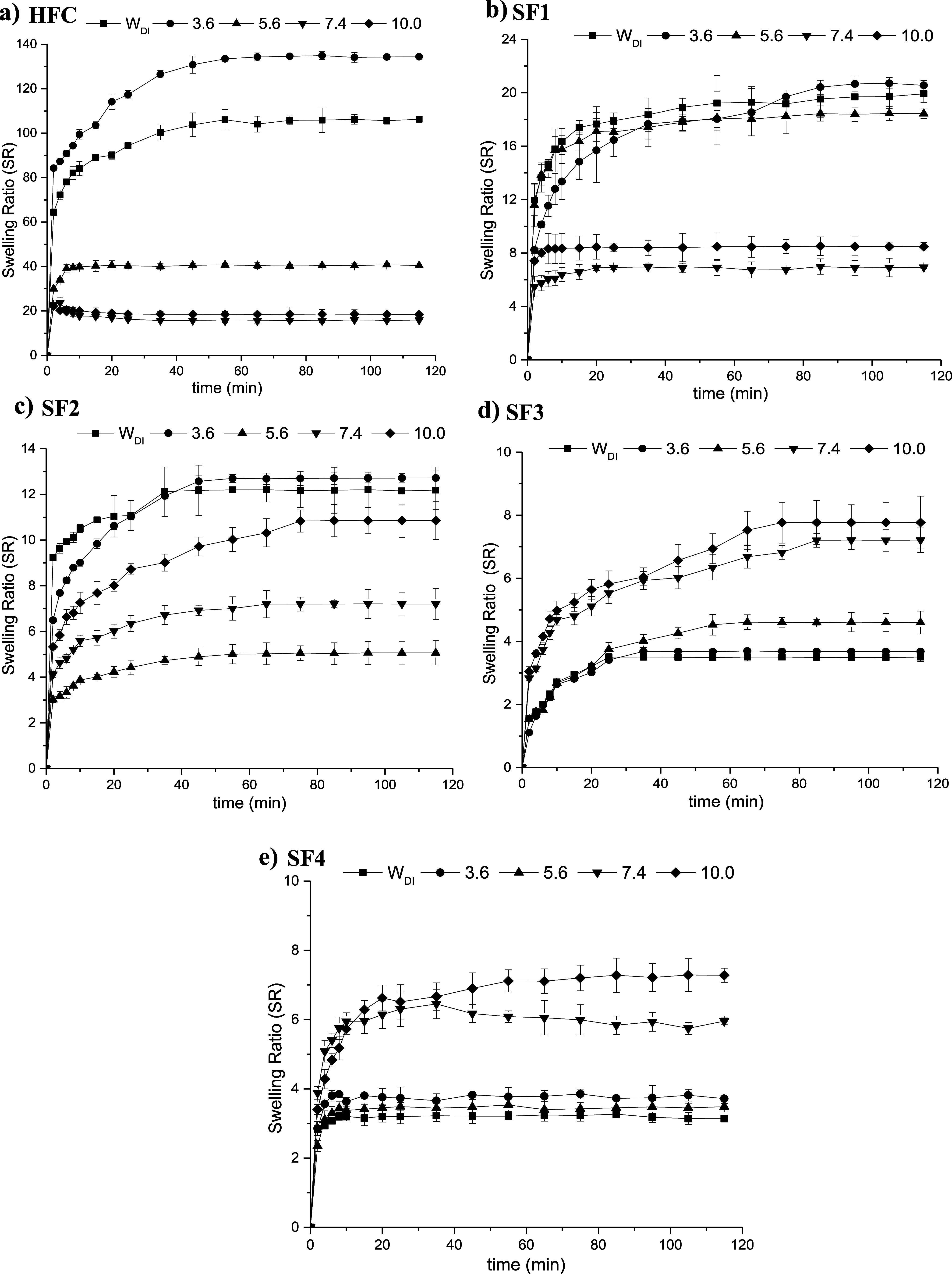
Swelling ratio of hydrogels in media of different pH at 37 °C; (a) HFC, (b) SF1, (c) SF2, (d) SF3, and (e) SF4, respectively.

**Figure 8:**
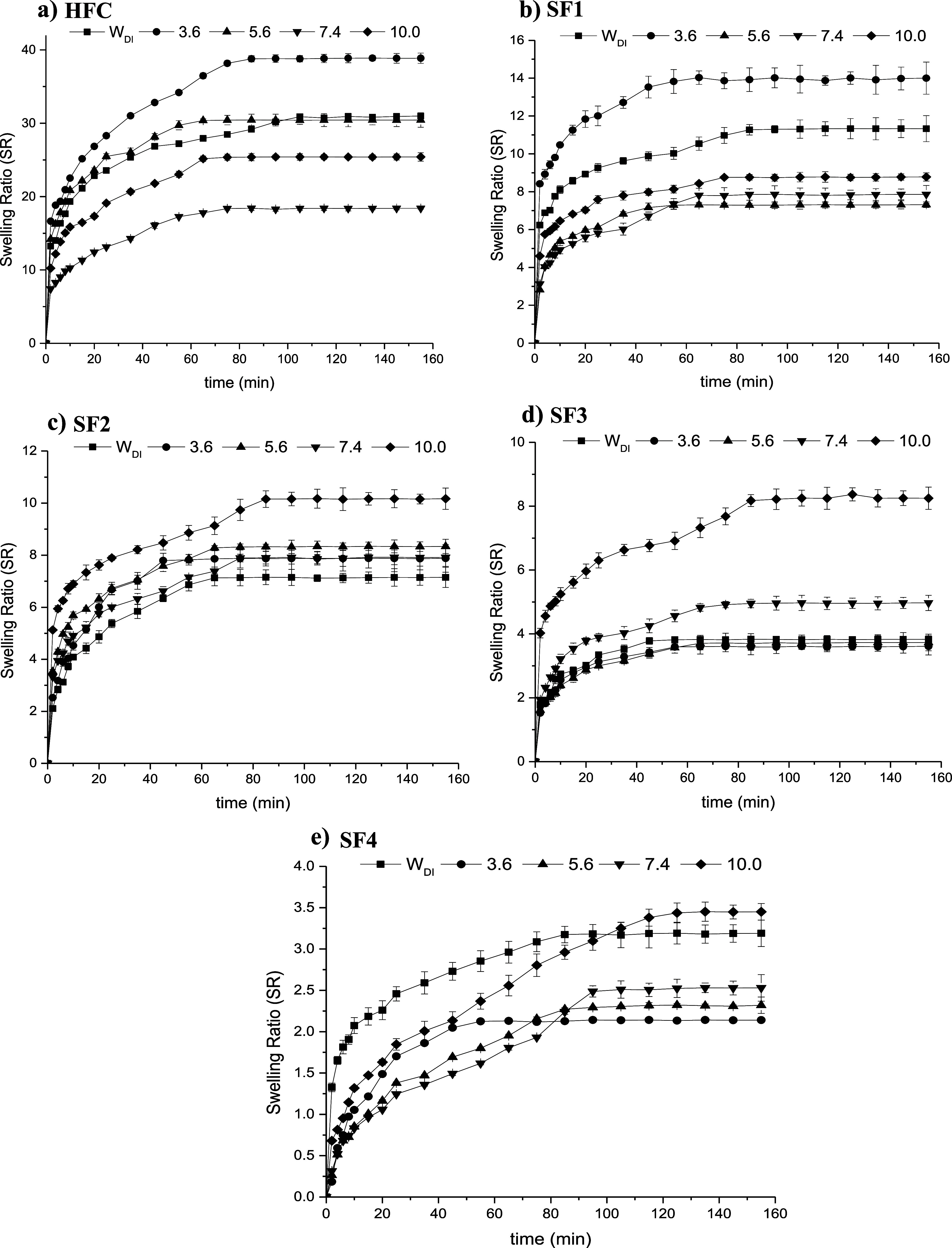
Swelling ratio of hydrogels in media of different pH at 25 °C; (a) HFC, (b) SF1, (c) SF2, (d) SF3, and (e) SF4, respectively.

**Table 3:** Hydrogels’ Swelling Capacity at Different pH and Temperature Conditions

	swelling ratio					
hydrogel	temp. (°C)	water _D.I._	pH = 3.6	pH = 5.6	pH = 7.4	pH = 10.0
**HFC**	25	30.99 ± 0.43	38.87 ± 0.7	30.44 ± 0.98	18.4 ± 0.13	25.41 ± 0.57
	37	106.27 ± 0.88	134.4 ± 0.5	40.41 ± 0.32	15.86 ± 0.08	18.41 ± 0.47
**SF1**	25	11.33 ± 0.69	14 ± 0.85	7.31 ± 0.15	7.86 ± 0.47	8.78 ± 0.22
	37	19.91 ± 0.63	20.55 ± 0.36	18.43 ± 0.35	6.92 ± 0.08	8.47 ± 0.3
**SF2**	25	7.15 ± 0.39	7.88 ± 0.53	8.34 ± 0.27	7.92 ± 0.35	10.17 ± 0.41
	37	12.18 ± 0.84	12.71 ± 0.48	5.06 ± 0.53	7.2 ± 0.67	10.85 ± 0.83
**SF3**	25	3.83 ± 0.11	3.61 ± 0.27	3.73 ± 0.26	4.97 ± 0.24	8.25 ± 0.35
	37	3.5 ± 0.13	3.68 ± 0.07	4.6 ± 0.36	7.21 ± 0.39	7.77 ± 0.84
**SF4**	25	3.19 ± 0.16	2.14 ± 0.01	2.32 ± 0.1	2.53 ± 0.16	3.45 ± 0.1
	37	3.13 ± 0.06	3.71 ± 0.03	3.48 ± 0.09	5.95 ± 0.09	7.28 ± 0.2

Based on the effect of pH, chitosan hydrogel has greater swelling capacity at lower pH, reaching the highest values at pH = 3.6. This behavior is because chitosan has a p*K*_a_ ∼ 6.5, so at lower pH, the greatest proportion of the amino groups in the matrix will be protonated, which generates electrostatic repulsion between them, so the chains to stabilize, they will tend to separate, which favors the diffusion of water into the hydrogel, increasing its swelling capacity. In semi-IPNs, incorporating γ-PGA into the chitosan hydrogel matrix generates a cross-linking effect due to the interactions established between the amino and carboxyl groups (neutral and protonated). Therefore, as there are more inter- and intrapolymeric interactions, there are fewer free sites to interact with the swelling medium. In general, the swelling capacity of hydrogels decreases with the formation of semi-IPNs, and this effect is more marked with the increase in the γ-PGA content. The swelling values for the semi-IPNs that have lower γ-PGA content (SF1 and SF2) are relatively higher at low pH, while the semi-IPNs that have a higher γ-PGA content (SF3 and SF4) have a greater swelling capacity at higher pH. This behavior is because γ-PGA has a p*K*_a_ = 4–4.8, so at higher pH values, the –COOH groups will be mostly in their ionized form. The electrostatic repulsion of the –COO^–^ groups will cause the separation of the chains, favoring the absorption of the aqueous solution and, consequently, increasing the swelling capacity of the hydrogels.^48,60–62^

### 3.8 In Vitro Cytotoxicity Assay

The graphs of the results of the cytotoxicity test are shown in Figure 9. As established by the ISO 10993-5 2009 standard, those materials that reduce cell viability by more than 30% are considered a cytotoxic effect. The extract of the individual chitosan hydrogel was the one with the lowest viability, which can be attributed to the fact that traces of acetic acid could have remained in the hydrogel, altering the pH of the medium and affecting the viability of the cells. It is recommended to perform an additional wash on the chitosan hydrogel to eliminate traces of acetic acid. SF1, SF2, and SF3 did not present a cytotoxic effect on Day 1. The semi-IPNs SF1 and SF2 were the hydrogels that showed the best cell viability and did not present a cytotoxic effect during the 3 consecutive days. The cell viability values reported for SF1 and SF2 during the 3 days do not present significant differences with the control. The improvement in cell viability can be attributed to the increase in the hydrophilicity of the semi-IPNs with the presence of γ-PGA in a lower proportion.^41^ Furthermore, the biocompatibility of γ-PGA hydrogels has been reported, associated with the presence of carboxyl groups that play an important role in cell adhesion.^63^ The semi-IPNs with a higher γ-PGA content presented lower viability values, which can be attributed to the fact that there is greater interaction between the polymers. The hydrogel matrix can encapsulate and retain nutrients that will be in deficit in the medium and that are necessary for the viability of the cells. For all samples, the four dilutions of each of the extracts showed cell viability values above 90%, that is, none of the dilutions presented a cytotoxic effect.

**Figure 9:**
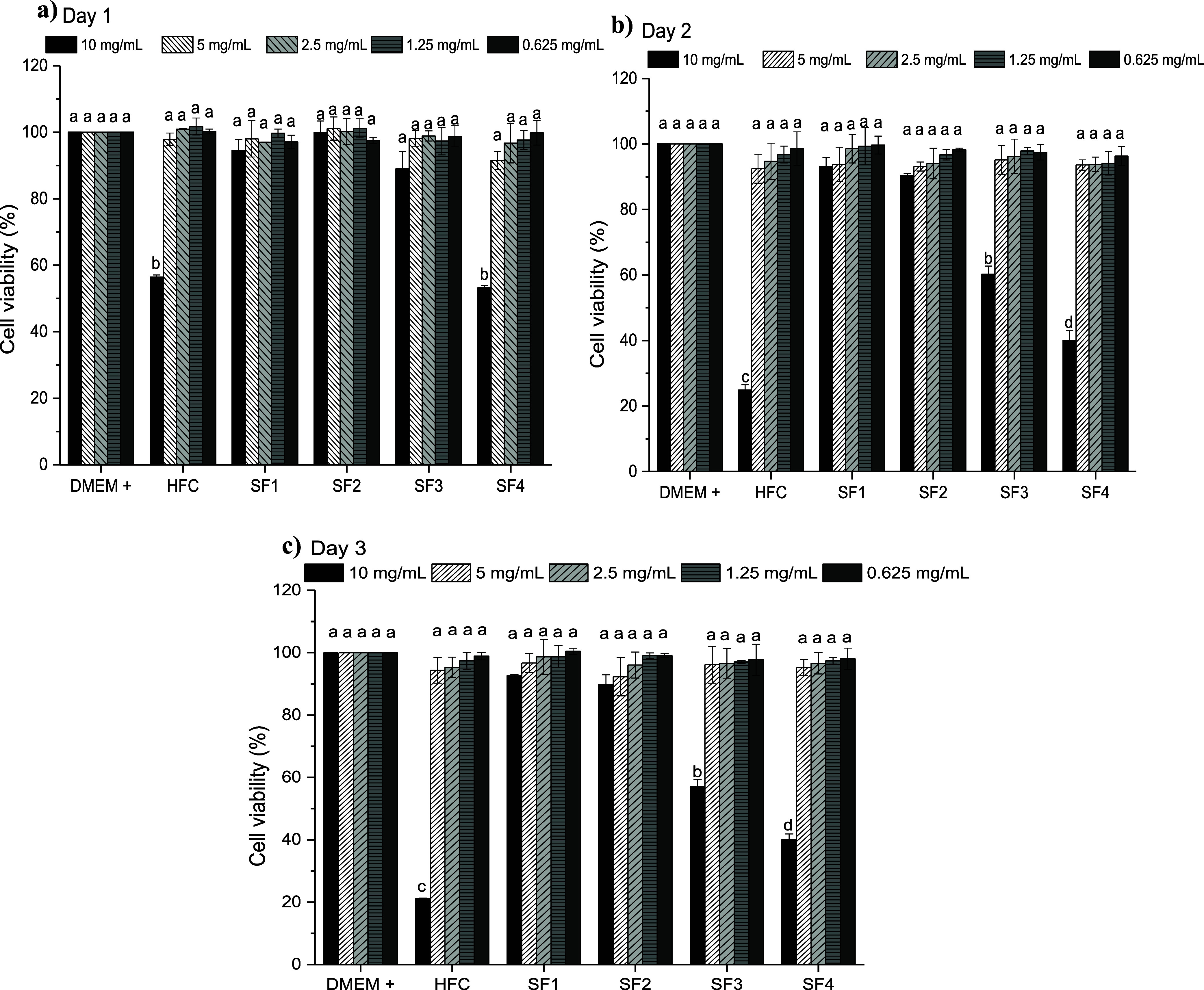
Cell viability (%) of the hydrogels at (a) day 1, (b) day 2 and (c) day 3.

## 4 Conclusions

A chitosan hydrogel was prepared by heating in an autoclave and subsequent freezing-thawing, to which the γ-PGA was incorporated. The FTIR spectra confirmed the presence of the functional groups of each polymer and the shifts of the signals resulting from the physical interactions between them. The SEM images revealed a porous structure of the hydrogels, which became denser and more compact with increasing γ-PGA content. This behavior was corroborated with the porosity test, which decreased with the formation of the reinforced network, with the pore density being lower with the increase in γ-PGA. The swelling capacity of hydrogels demonstrates their sensitivity to pH and temperature. For semi-IPNs hydrogels, SF1 had the highest swelling ratio (20.55) at pH 3.6 and *T* = 37 °C, while the lowest value was reported for SF4 (2.14) at pH 3.6 and *T* = 25 °C. With the formation of the semi-IPN, the swelling capacity of the hydrogels decreased, due to greater interaction between both polymers concerning their interaction with the swelling medium. The formation of the semi-IPNs brought about improvements in the mechanical properties, with respect to the simple Chitosan hydrogel, improving the resistance with the increase in the γ-PGA content. The cell viability assay demonstrated that the presence of γ-PGA contributed to improving the biocompatibility of the materials, obtaining the best results for the semi-IPNs SF1 and SF2, for three consecutive days. These results suggest that the semi-interpenetrated Chitosan/γ-PGA networks obtained may be promising materials with great potential to be used in biomedical applications.
